# Charity Hospital Training School for Nurses

**Published:** 1893-07

**Authors:** 


CHARITY HOSPITAL TRAINING SCHOOL FOR NURSES, NEW ORLEANS.—The main purposes of this school are to educate and suitably train classes of good and intelligent women, who may follow their vocation as trained nurses in the Charity Hospital or elsewhere. Young women of good character who desire to learn a good profession, should write to Sister Agnes, Charity Hospital, New Orleans, for blanks and instructions.

This is another avenue open to women, where they can make a support, and at the same time do good. The services of a trained professional nurse are always in demand in cities, and they receive good wages. Those who do not want to teach or sew or clerk in a shop, will do well to investigate this opportunity.

